# Neurodevelopment in the third year of life in children with antenatal ZIKV-exposure

**DOI:** 10.11606/s1518-8787.2021055002798

**Published:** 2021-04-08

**Authors:** Carolina Yuri Panvequio Aizawa, Deyse Mayara Rodrigues Caron, Carolina Barbosa de Souza, Paula Fernanda Augusto Kozima, Luana Damasceno, Christa Einspieler, Peter B. Marschik, Patrícia Brasil, Ana Carolina Basso Schmitt, Karin Nielsen-Saines, Renata Hydee Hasue

**Affiliations:** I Universidade de São Paulo Faculdade de Medicina São PauloSP BR Universidade de São Paulo. Faculdade de Medicina. Programa de Pós-Graduação em Ciências da Reabilitação. São Paulo, SP, BR; II Universidade de São Paulo Faculdade de Medicina Departamento de Fisioterapia São PauloSP BR Universidade de São Paulo. Faculdade de Medicina. Departamento de Fisioterapia, Fonoaudiologia e Terapia Ocupacional. São Paulo, SP, BR; III Fundação Oswaldo Cruz Instituto Nacional de Infectologia Evandro Chagas Rio de JaneiroRJ BR Fundação Oswaldo Cruz – FIOCRUZ. Instituto Nacional de Infectologia Evandro Chagas. Rio de Janeiro, RJ, BR; IV Medical University of Graz Division of Phoniatrics. iDN – interdisciplinary Developmental Neuroscience Graz Austria Medical University of Graz. Division of Phoniatrics. iDN – interdisciplinary Developmental Neuroscience. Graz, Austria; V University Medical Center Göttingen & Leibniz ScienceCampus Primate Cognition Child and Adolescent Psychiatry and Psychotherapy Göttingen Germany University Medical Center Göttingen & Leibniz ScienceCampus Primate Cognition. Child and Adolescent Psychiatry and Psychotherapy. Göttingen, Germany; VI Karolinska Institute Center of Neurodevelopmental Disorders (KIND) Department of Women's and Children's Health Stockholm Sweden Karolinska Institute. Center of Neurodevelopmental Disorders (KIND), Department of Women's and Children's Health. Stockholm, Sweden; VII University of California Los Angeles David Geffen School of Medicine Department of Pediatrics Los AngelesCA USA University of California Los Angeles. David Geffen School of Medicine. Department of Pediatrics. Los Angeles, CA, USA

**Keywords:** Child Development, Language Development, Cognition, Motor Activity, Zika Virus

## Abstract

We report cognitive, language and motor neurodevelopment, assessed by the Bayley-III test, in 31 non-microcephalic children at age 3 with PCR-confirmed maternal Zika virus exposure (Rio de Janeiro, 2015–2016). Most children had average neurodevelopmental scores, however, 8 children (26%) presented delay in some domain. Language was the most affected: 7 children (22.6%) had a delay in this domain (2 presenting severe delay). Moderate delay was detected in the cognitive (3.2%) and motor (10%) domains. Maternal illness in the third trimester of pregnancy and later gestational age at birth were associated with higher Bayley-III scores. Zika-exposed children require long-term follow-up until school age.

## INTRODUCTION

Zika virus (ZIKV) can damage the developing brain of infants with antenatal exposure to acute maternal illness with complications leading to fetal or infant death. Although congenital microcephaly is the most dramatic sequela, Congenital Zika Syndrome (CZS) includes a wide range of neurological abnormalities even in non-microcephalic infants, such as seizures, hypertonia/hypotonia, ataxia, dyskinesia, irritability, abnormal posturing and movements, as well as auditory and visual deficits. Many of these findings are associated with delayed neurodevelopment^1^.

Monitoring the growth and development of ZIKV-exposed infants has global relevance for adequate clinical diagnosis and management, as well as for implementation of public health policies. Microcephalic infants with CZS show severe delay and atypical neurodevelopment during their first years of life^1^, while little is known regarding asymptomatic non-microcephalic children with antenatal ZIKV exposure. The most recent analysis of 216 ZIKV-exposed children from the Oswaldo Cruz Foundation (Fiocruz), Rio de Janeiro, Brazil, revealed below-average neurodevelopment and/or abnormal eye or hearing assessments in 31.5% of children between 7 to 32 months of age^2^.

Considering that repercussions of antenatal infection may appear later in childhood, it is important to follow up neurodevelopment as children grow older. The current gold standard method for neurodevelopment analysis are the Bayley Scales of Infant and Toddler Development, Third Edition (Bayley-III), which are accepted as a valid and reliable measure for children between 1 and 42 months of age and has been used for many years in different populations, for example, in preterm children^3^^,^^4^. Continuous monitoring of neurodevelopment in the present cohort enables drafting of improved health care strategies within the Brazilian Unified Health System. Therefore, the main purpose of this study was to assess cognitive, language and motor development in 31 non-microcephalic children, antenatally exposed to ZIKV, aged 3 years or older from the Fiocruz cohort^2^.

## METHODS

Thirty-one non-microcephalic infants (54.8% male; mean gestational age = 37.9w, SD = 1w 5d) antenatally exposed to ZIKV infection were assessed for cognitive, receptive and expressive language, as well as fine and gross motor development, with the Bayley-III scale at around 3 years (33–38 months; mean = 36, SD = 1,24). All pregnant women had symptomatic illness with a rash during the 2015–2016 Rio de Janeiro outbreak, and positive Reverse Transcription Polymerase Chain Reaction (RT-PCR) results in blood and/or urine, confirming ZIKV antenatal infection. Other etiologies were ruled out [TORCH (toxoplasma gondii, Treponema pallidum, rubella, cytomegalovirus, herpes simplex), Varicella-zoster virus, Human Immunodeficiency virus (HIV), congenital anomalies, prenatal substance use].

The Ethics Committee of the University of São Paulo and Fiocruz (Protocol CAPPESQ 135/16; CAAE: 55685416.9.3001.5262) approved the study. Written informed consent was obtained from parents of all children enrolled.

Two licensed physiotherapists conducted Bayley-III assessments in a quiet 4 × 2 × 5 m sized room, in the presence of at least one parent, at Fiocruz, Manguinhos, Rio de Janeiro, Brazil, between November 2018 and July 2019. Moderate developmental delay in a respective domain of Bayley-III was defined as composite scores < 85 (< −1SD); severe developmental delay as composite scores < 70 (< −2SD)^3^. Bayley-III categories were applied to identify children's development as ‘well above average’, > 2SD (score > 131); ‘above average’, 1-2SD (score 116-130); ‘average’, −1 to 1SD (score 85-115); ‘below average’, −1 to −2SD (score 84–70); ‘well below average’, < −2SD (score < 70)^2^.

Clinical and socioeconomic characteristics were evaluated for possible associations with Bayley-III domains: gender, trimester of maternal infection (1^st^, 2^nd^, 3^rd^ trimester), gestational age at birth (weeks), birth weight (grams), Apgar scores (1^st^ and 5^th^ minute), parental schooling level (basic, secondary, higher education) and family income (ranging from 1 to 5 minimum wages). Clinical data was collected from medical records; socioeconomic parameters were obtained from parents.

Bivariate analysis was performed to understand influences of clinical and socioeconomic variables on Bayley-III composite and scaled scores among ZIKV-exposed non-microcephalic children. For categorization of the dependent variable, Bayley-III, medians were used as cut-off points for both scores when few or no observations were obtained below the 85-cut-off point. Software STATA for MacBook, version 14.2, was used for statistical analysis, with p ≤ 0.05 indicating statistical significance.

## RESULTS

Clinical, socioeconomic and neurodevelopmental characteristics of the 31 children are shown in Table 1. Eight children (26%) scored below average in at least one Bayley-III domain. The language domain was the most affected (n = 7, 22.6%), revealing moderate delay in five children (scores < 85–70) and severe delay in two children (scores < 70). Moderate cognitive delay was found in one child (3.2%) and below average motor function in three children (10%). Case 5 had moderate delay in all domains; Case 2 had moderate delay in the language and motor domains. The two preterm children assessed (Cases 1 and 9) had lower scores in language development (Case 1 showed severe delay).

**Table 1:** Clinical, socioeconomic and neurodevelopmental assessments of antenatally ZIKV-exposed non-microcephalic children at three years of age by Bayley Scales of Infant and Toddler Development, Third Edition.

Case no.	Gender	Trimester of maternal infection	GA at birth (weeks)	BW (g)	Apgar 1min/5min	Mother's education	Father's education	Family income (m. wage)	Bayley-III Composite score/classification^a^		
									Cognitive	Language	Motor
1	F	2	35	2,004	6/8	SE	SE	2–3	85/C	59/E	85/C
2	M	3	39	3,240	9/10	HE	HE	> 5	90/C	77/D	76/D
3	F	3	40	4,190	9/9	SE	SE	3–4	100/C	83/D	154/A
4	M	2	38	2,825	9/9	BE	BE	2–3	90/C	83/D	97/C
5	M	1	36	3,220	NI	SE	SE	> 5	75/D	77/D	82/D
6	M	1	38	3,605	8/9	SE	SE	3–4	85/C	62/E	94/C
7	F	1	36	2,700	9/9	SE	BE	2–3	90/C	83/D	94/C
8	M	2	37	3,185	9/9	SE	SE	2–3	95/C	89/C	82/D
9	F	1	31	1,430	9/10	SE	SE	2–3	90/C	89/C	97/C
10	F	3	37	2,750	NI	SE	SE	> 5	105/C	106/C	112/C
11	F	3	39	3,220	9/10	SE	SE	3–4	100/C	124/B	103/C
12	M	3	38	3,745	9/9	SE	SE	3–4	140/A	103/C	121/B
13	F	2	38	3,735	9/9	SE	SE	> 5	100/C	124/B	94/C
14	M	2	40	2,885	9/9	SE	SE	> 5	110/B	103/C	103/C
15	M	3	38	3,120	9/10	SE	SE	2–3	95/C	103/C	100/C
16	M	2	38	3,975	9/9	SE	SE	2–3	95/C	121/B	100/C
17	F	2	40	3,795	9/10	HE	HE	> 5	100/C	106/C	107/C
18	F	2	37	3,350	8/9	SE	SE	1–2	110/B	97/C	103/C
19	M	2	35	2,995	8/9	HE	HE	> 5	115/B	89/C	85/C
20	M	2	39	3,610	2/5	SE	SE	3–4	100/C	97/C	100/C
21	M	2	38	3,400	10/10	HE	HE	> 5	100/C	109/C	88/C
22	M	2	39	3,685	8/10	SE	SE	4–5	105/C	106/C	110/C
23	F	2	38	3,275	8/10	SE	SE	3–4	100/C	112/C	100/C
24	F	3	38	3,200	NI	SE	SE	> 5	95/C	106/C	107/C
25	M	1	38	3,220	9/9	SE	BE	2–3	105/C	106/C	130/B
26	M	3	39	3,640	8/10	HE	HE	> 5	100/C	100/C	100/C
27	F	2	39	3,850	NI	HE	HE	> 5	100/C	94/C	100/C
28	F	3	37	2,765	9/10	SE	BE	3–4	100/C	103/C	115/C
29	M	3	39	2,935	9/10	HE	BE	3–4	100/C	106/C	112/C
30	M	3	NI	3,520	8/9	SE	SE	2–3	95/C	94/C	103/C
31	F	3	39	3,286	NI	SE	SE	3–4	105/C	106/C	107/C

Bayley-III: Bayley Scales of Infant and Toddler Development III; BE: basic education; BW: birth weight; Ext.Low: extremely low; F: female; g: grams; GA: gestational age; H. Average: high average; HE: higher education (higher parental level of education is more than 12 years in school); L. Average: low average; M: male; min: minute; m. wage: minimum wage (R$998.00); N: normal; NA: not assessed; NI: not informed; SE: secondary education; V. Superior: very superior; ZIKV: zika virus.

a Composite score performance categories: A: well above average, > 2 SD (score > 131); B: above average, 1–2 SD (score 116–130); C: average, −1 to 1 SD (score 85–115); D: below average, −1 to −2 SD (score 84–70); E: well below average, < −2SD (score < 70). ^2^ Scores below −1 SD are highlighted.

Brainstem Evoked Response Audiometry (BERA) was performed on 17 of the 31 children, all with normal results. Among the eight who scored below average in language domain, five (Cases 1, 3, 4, 7, 8) presented normal BERA results while three did not perform the test.

Infection in the third trimester of pregnancy was associated with higher Bayley-III scores in the motor domain (OR = 0.02; 95%CI 0.01–0.45), especially gross motor function (OR = 0.05; 95%CI 0.01–0.72) (Table 2). Prematurity showed significant associations with below average language (OR = 15.00; 95%CI 1.53–146.54) and motor (OR = 10.2; 95%CI 1.55–67.21) results, especially considering gross motor function (OR = 15.00; 95%CI 1.53–146.54) (Table 2). No statistically significant associations were seen between the cognitive domain with trimester and gestational age; and for any Bayley-III domain with gender, birth weight, Apgar scores, parental education and family income.

**Table 2:** Bivariate analysis of clinical and socioeconomic factors with Bayley-III scores among antenatally ZIKV-exposed non-microcephalic children (n = 31) at 3 years of age.

	Gross motor domain			Motor domain			Language domain		
	n (%)	OR (95%CI)	p	n (%)	OR (95%CI)	p	n (%)	OR (95%CI)	p
Trimester 1o	4 (28.6)	ref.		4 (36.4)	ref.		–	–	
2o	8 (57.1)	0.33 (0.03–3.80)	0.376	6 (54.5)	0.19 (0.01–2.14)	0.178	–	–	
3o	2 (14.3)	0.05 (0.01–0.72)	0.028	1 (9.1)	0.02 (0.01–0.45)	0.13	–	–	
Prematurity (< 260 days)	7 (50.0)	15.00 (1.53–146.54)	0.020	6 (54.5)	10.2 (1.55–67.21)	0.016	7 (50.0)	15.00 (1.53–146.54)	0.020

Bayley-III: Bayley Scales of Infant and Toddler Development III; OR: odds ratio; 95%CI: 95% confidence interval; ref.: reference.

## DISCUSSION

The majority of our non-microcephalic children exposed to antenatal ZIKV infection had composite scores above 85 on Bayley-III cognitive and motor domains (96.8% and 90%, respectively), suggesting a predominantly normal neurodevelopmental outcome in the third year of life. However, scores below average in any Bayley-III domain were identified in 26% of children, mostly for the language domain. Language delay was observed in 22.6% of cases, including two children with severe cases.

The main concern following antenatal ZIKV exposure is whether clinical manifestations appear later in life in non-microcephalic infants. Our recent analysis^2^ of 146 children aged between 7 and 32 months revealed that 40% of them had developmental delay in at least one Bayley-III domain, with language function being the most affected^2^. In this smaller case series, we observed the same pattern of developmental delay in ZIKV-exposed children at the age of three.

Congenital infection or exposure to other viruses can also lead to development delay during preschool years^5^^,^^6^. Later in life, poor language ability is associated with retention in school and the necessity to attend special education classes for children exposed to HIV^5^, and with expressive language delay (even without hearing loss) for children with congenital cytomegalovirus infection. Poorer school performance and lower quality of life with long-term impairment are also present^6^. Although some of our children had normal BERA test results, we found language delay in eight of them. Taken together, these data reinforce the need of maternal-infant health policies to effectively prevent congenital infections and adequately manage children at risk.

Maternal illness during the third trimester of pregnancy was associated with higher Bayley-III scores, and prematurity was associated with lower Bayley-III scores, as reported in our prior study^2^. These findings corroborate the assertion that earlier infection during gestation correlates with more severe brain involvement and dysfunction. Since ZIKV infection may also predispose preterm birth, the effects on pediatric neurodevelopmental outcomes may be both from direct (timing of central nervous system development during gestation) and indirect (prematurity) consequences of ZIKV exposure *in utero*.

In contrast to language, most of our children scored > 85 for the cognitive domain (96.8%). However, the affinity of ZIKV for the nervous system structures responsible for cognitive function was demonstrated in animal models^7^. Given that this is a newly identified congenital illness, it is not possible to affirm that neurocognitive function in ZIKV-exposed children will be intact throughout their future. Language delay in fact may predict increased risk of developmental delays and learning difficulties at school age. The percentage of our children with motor delay (10%) was lower than those with language delay (22.6%), which may be related to the early physical therapy intervention in children with abnormal neurological examinations (61% of the cohort) between the ages of 1 to 3 months, contributing to better performance in motor scores.

Our main limitations included the small sample size, related to difficulties in retaining seemingly unaffected children in long-term follow-up. Not performing confirmatory audiometric evaluations in all children was another limitation, even with no visual or hearing abnormalities detected on early screening. Although ophthalmologic abnormalities have not been shown to progress in congenital ZIKV infection, little is known regarding development or progression of hearing deficits over time^8^. Although we used the Brazilian validated version of Bayley-III, we consider a limitation of this study the absence of a control group with similar socioeconomic conditions in order to compare baseline rates of cognitive, language and motor normal development in a contemporaneous non-ZIKV-exposed sample. Nevertheless, the risk of bias during Bayley-III assessment was minimized since the examiners were not aware of the results of ZIKV RT-PCR confirming or not maternal infection.

Our study provides important information about the spectrum of neurodevelopmental outcomes in children at age three who had antenatal ZIKV exposure, and it brings attention to long-term follow-up of non-microcephalic infants. Our results show a high percentage of typical neurodevelopment, but also demonstrate that adverse outcomes can be identified. There is still need for further investigation on possible long-term delay and neurologic sequelae until school age, beyond evaluations of the motor domain. Intersectoral public policies to prevent and manage maternal infections, also aimed at promoting neurodevelopmental surveillance and early interventions for affected children, are necessary to minimize learning disabilities during childhood, that can often impact quality of life and reduce opportunities.
